# Making sense of movement in embodied design for mathematics learning

**DOI:** 10.1186/s41235-016-0034-3

**Published:** 2016-12-19

**Authors:** Dor Abrahamson, Arthur Bakker

**Affiliations:** 1 Graduate School of Education, University of California, Berkeley, 4649 Tolman Hall, Berkeley, CA 94720-1670 USA; 2 Utrecht University, Utrecht, The Netherlands

**Keywords:** Attentional anchor, Ecological dynamics, Embodiment theory, Enactivism, Interaction, Eye tracking, Mathematical imagery trainer, Mathematics, Tablet, Technology

## Abstract

Embodiment perspectives from the cognitive sciences offer a rethinking of the role of sensorimotor activity in human learning, knowing, and reasoning. Educational researchers have been evaluating whether and how these perspectives might inform the theory and practice of STEM instruction. Some of these researchers have created technological systems, where students solve sensorimotor interaction problems as cognitive entry into curricular content. However, the field has yet to agree on a conceptually coherent and empirically validated design framework, inspired by embodiment perspectives, for developing these instructional resources. A stumbling block toward such consensus, we propose, is an implicit disagreement among educational researchers on the relation between physical movement and conceptual learning. This hypothesized disagreement could explain the contrasting choices we witness among current designs for learning with respect to instructional methodology for cultivating new physical actions – whereas some researchers use an approach of direct instruction, such as explicit teaching of gestures, others use an indirect approach, where students must discover effective movements to solve a task. Prior to comparing these approaches, it may help first to clarify key constructs. In this theoretical essay we draw on embodiment and systems literature as well as findings from our design research so as to offer the following taxonomy that may facilitate discourse about movement in STEM learning: (1) distal movement is the technologically extended effect of physical movement on the environment; (2) proximal movement is the physical movements themselves; and (3) sensorimotor schemes are the routinized patterns of cognitive activity that become enacted through proximal movement by orienting on so-called attentional anchors. Attentional anchors are goal-oriented phenomenological objects or enactive perceptions (“sensori-”) that organize proximal movement to effect distal movement (“-motor”). All three facets of movement must be considered in analyzing embodied learning processes. We demonstrate that indirect movement instruction enables students to develop new sensorimotor schemes including attentional anchors as idiosyncratic solutions to physical interaction problems. These schemes are, by necessity, grounded in students’ own agentive relation to the world while also grounding target content such as mathematical notions.

## Significance

Engineering developments in computational technology have created unprecedented opportunities for industry to build and disseminate mathematics-education applications (“apps”). Thousands of these apps are now literally at the fingertips of any child who can access a tablet, smartphone, or personal computer with a responsive touchscreen. Educational researchers could contribute to the quality of these ubiquitous consumer products by offering design frameworks informed by theories of learning. However, existing frameworks are derived from interaction theories drawing on epistemological assumptions that are no longer tenable, given the embodiment turn in the cognitive sciences. A proposed systemic reconceptualization of mathematical objects as grounded in sensorimotor schemes for material interaction offers educational designers heuristics for creating activities in which students learn by discovering motion patterns.

*“All that is important is this one moment in movement. Make the moment important, vital, and worth living. Do not let it slip away unnoticed and unused”* (Martha Graham).

## Background

### Embodiment rising

In recent decades, we have witnessed a collective reevaluation of what we know about the human cognitive architecture (Núñez & Freeman, 1999). Metaphors of the mind as a central processing unit are all but gone, making room for alternative epistemological conceptualizations (Kiverstein, 2012). One intriguing set of proposals, loosely referred to as embodiment theory, offer views of the mind as extending out of the head, through the body, and into the natural and sociocultural ecology (Anderson, 2003; Wilson, 2002; Yanchar, Spackman, & Faulconer, 2013). By these views, which may vary widely in their commitments and details, mind and body are not separate entities but instead form an irreducible ontology, wherein sensorimotor activity is intrinsic to learning, knowing, and reasoning. Furthermore, some authors advance the perspective that human behavior in social ecologies is best modeled from a systemic perspective that subsumes multiple individuals interacting in complex activity structures regulated by cultural forms that are themselves constantly evolving (Malafouris, 2013; Melser, 2004).

Our research program aspires to advance and refine embodiment theory through investigating how it may benefit the educational enterprise. We are particularly interested in understanding relations between physical actions and conceptual learning as these relations bear on theoretical and pragmatic problems in the research field of mathematics education and perhaps beyond into other STEM domains. This theoretical paper attempts to contribute to the research discourse on embodiment as it pertains to mathematics education. We will be offering analytic constructs for speaking about physical action in ways that could inform the practice of educational design research, that is, the science of building effective instructional resources. In particular, we will offer a taxonomy of movement that, we hope, could lead to empirical work evaluating best instructional methodology for action-based learning.

### The primacy of movement

Embodiment theory rejects fundamental tenets of Cartesian dualism, the dominant historical epistemology. According to the Cartesian view, the enfleshed body is an input/output conduit for the brain – physical actions execute cerebral commands, while perceptual faculties, predominantly vision, guide and monitor these actions, collecting for the brain information on the results of the actions. The Cartesian mechanism bears intuitive explanatory appeal, which may explain its historical resilience, and yet the model has been increasingly challenged from diverse fields of scholarship, including philosophy (Gallagher, 2015; Gangopadhyay & Kiverstein, 2009; Merleau-Ponty, 1964; Noë, 2006; Sheets-Johnstone, 1981), cognitive psychology (Barsalou, 2010; Witt & Riley, 2014), cognitive development (Lozada & Carro, 2016; Marshall, 2016; Thelen & Smith, 1994), dynamical systems (Kauffman, 1995; Kelso, 1995; Turvey, 1992), human–computer interaction (Dourish, 2001; Gillies & Kleinsmith, 2014), and robotics (Clark, 1999). Physical movement, these critical scholars believe, is not the executive arm of an abstracted intelligence. Rather, moving is situated in dynamical cognition. Moving marks adaptive, self-organizing, goal-oriented systemic intelligence in growth (Kelso, 2000).

This “primacy of movement” (Sheets-Johnstone, 1999) is the formative human condition and thus includes learning and reasoning across disciplines, contexts, and media. Cognition develops in streaming activity of ecologically embodied, embedded, and distributed interaction. Cognitive activity may be actual, projected, or even imagined, as when we sit still with our eyes closed. Even then, our conscious experience of inner sensory perceptions and dialogue need not imply a contentful mind. Rather, some scholars believe that mental representations, which have been a focal historical construct of cognitive science, are for the most conscious epiphenomena of an intrinsically enactive mind at work (Chemero, 2009; Hutto & Myin, 2013; Varela, Thompson, & Rosch, 1991).

Our own position is much related to enactivism. We are inspired by the following words from Varela et al. (1991), where they summarize the emergence of concepts from the development of sensorimotor skill:

“[T]*he enactive approach consists of two points: (1) perception consists in perceptually guided action; and (2) cognitive structures emerge from the recurrent sensorimotor patterns that enable action to be perceptually guided*” (p. 173).

Where we use the verb “to enact” as well as its conjugations and cognates, we intend it as content-agnostic – by saying that a person enacted some particular movement, whether physically or imaginatively, we deliberately wish to avoid ascribing or not to that movement epistemic appendages such as understanding, meaning, intention, or grounding. We hope this use of the verb will help to clarify our position and arguments.

### Embodiment in educational design research

The embodiment turn in the cognitive sciences has been of considerable interest to educational researchers, as evidenced in an accumulating body of literature seeking to understand the implications of this philosophical turn for pedagogical theory and practice (Abrahamson, in press; Begg, 1999; Davis & Sumara, 2008; Hall & Nemirovsky, 2012; Hutto, Kirchhoff, & Abrahamson, 2015; Kieren, Gordon Calvert, Reid, & Simmt, 1995). Embodiment theory’s proposed centrality of sensorimotor activity in human learning found fertile grounds in educational scholarship, where seminal ideas from pragmatism (Dewey, 1916/1944), constructivism (Piaget, 1968), and cultural–historical theory (Vygotsky, 1926/1997) had already articulated the formative role of situated interaction in cognitive development.

As the field of educational research turned its attention to physical actions performed in context, a wealth of educational studies ensued that documented and theorized the multimodal behaviors that people perform when they engage mathematical content, such as when they teach (Alibali et al., 2013), learn (Abrahamson, 2004; Goldin-Meadow, Wagner Cook, & Mitchell, 2009; Lemke, 2003; Radford, 2003), problem solve (Goldin-Meadow, Nusbaum, Kelly, & Wagner, 2001), and argue (Ochs, Gonzales, & Jacoby, 1996; Schwarz & Prusak, 2016). For the most, these studies have treated externally manifest physical movements. Yet, movements during mathematical activity may also be imaginary – introspective reports from both experts (Hadamard, 1945) and novices (Presmeg, 1998) suggest the role of imagination in mathematical inferential reasoning, and neuroscience experimentation concurs (Gallese & Lakoff, 2005; Zeki, Romaya, Benincasa, & Atiyah, 2014).

When individuals reason, their cognitive activity is implicitly embedded within sociocultural structures (Sawyer, 2007; Stetsenko, 2002). In particular, when people engage in mathematical discourse, they draw on a variety of personal and material resources to construct, depict, and explain their reasoning in the form of psychological objects and actions that would be intelligible to their interlocutors (Kirsh, 2013; Nemirovsky & Borba, 2004; Nemirovsky & Ferrara, 2009; Nemirovsky, Kelton, & Rhodehamel, 2012; Radford, 2014). These schematized multimodal expressions become the shared referents of a collective practice (Becvar, Hollan, & Hutchins, 2005; Lakoff & Núñez, 2000). In turn, these socially emergent cultural forms then regulate human activity, specifically individual reasoning (Malafouris, 2013; Saxe, 2012; Wertsch, 1979).

As we look to educational researchers’ conceptualizations of movement, we wish here further to narrow our focus onto the work of instructional designers and in particular design researchers. Design researchers (or design-based researchers) are educational researchers whose empirical studies are lodged in the practice of engineering, building, and evaluating learning environments (Bakker & Van Eerde, 2015; Cobb, Confrey, diSessa, Lehrer, & Schauble, 2003). A substantial number of design researchers have been inspired by embodiment theory to envision innovative platforms, materials, activities, and facilitation techniques that leverage the physical actions students perform as important resources for content learning (Lee, 2015; Malinverni, Ackermann, & Pares, 2016; Manches & O’Malley, 2016; Smith, King, & Hoyte, 2014). By and large, the objective of these designs is to create conditions for cultivating students’ enactment of particular motor actions that would presumably lead to understanding some targeted conceptual content. In one design, for example, students jump sidewise along a number-line mat in response to numerical cues flashed on a screen, thus grounding conceptions of relative numerosity into a spatial representation that is used pervasively in mathematical practice (Fischer, Moeller, Bientzle, Cress, & Nuerk, 2011).

Having briefly reviewed the rise of embodiment theory, the centrality of movement in the theory, and the application of the theory to educational research, we now turn to introduce a research problem that will serve us as a case study throughout this paper. We will then argue that embodiment theory could tackle this problem if the field agreed on analytic definitions of movement. The paper then offers these definitions and exemplifies their application.

### The paradox of learning to operate new mathematical objects

In activities such as the number-line mat, students receive direct instructions on how to move, and then the students practice moving accordingly. It seems logical that students should be directly instructed to move in patterns associated with expert mathematical practice. Indeed, across all human disciplines, arts, and crafts, novices are apprenticed by receiving explicit instructions on how to wield objects of their trade, be these a mason’s trowel, a plumber’s wrench, or a violinist’s bow (Ingold, 2000). In these manual practices, engaging tools effectively coevolves with developing expert perception both of the tools themselves and the domain they are applied to and generate (Goodwin, 1994; Vérillon & Rabardel, 1995). To emphasize, these tools and domains of the manual trades are perceptually accessible, externally manifest entities. Yet, how could direct movement instruction work in the discipline of mathematics, where novices do not perceive the tools and domains they are yet to learn? How can you operate on an object you do not yet see?

Mathematical objects, such as a proportion, are both like and unlike material objects. Similar to a trowel, wrench, or bow, a proportion is something people can legitimately talk about – it can exist in semiotic social space as a bonafide shared referent of multimodal discourse. Unlike material objects, however, mathematical objects are not manifest referents of the instructional environment (cf. Bakker & Hoffmann, 2005) but need to be co-constructed in discursive space (Nemirovsky et al., 2012). This tentative ontology of mathematical objects encumbers instructional conversations, which depend on some initial shared referent, even if the referent is ambiguous or still emerging into prospective discursive space (Flood, Harrer, & Abrahamson, 2016; Foster, 2011; Isaacs & Clark, 1987; Moschkovich, 2015; Newman, Griffin, & Cole, 1989; Sfard, 2002). As such, students entering embodied–interaction spaces face a double challenge – they cannot manipulate the object so as to satisfy the task specifications, and they cannot see the object in order to enact the manipulation and evaluate the efficacy of their actions. How might this learning paradox be unraveled?

We propose that undoing this paradox begins from reconceptualizing movement from embodiment perspectives as sensorimotor cognitive activity.^1^ From this view, it is not the case that students must discover both how to move (motor) and what to perceive (sensori-), they must discover how to move by discovering what to perceive (and vice versa). This enactivist proposal to reconceptualize the ontology of physical movement builds on cognitive developmental research as well as dynamical systems theories. As Piaget (1968, 1970) asserted, sensorimotor schemes are enacted routines comprising both what you operate on and how you operate on it. These complementary aspects of goal-oriented situated action co-evolve reciprocally and recursively through mutual adaptation into systemic functioning structures (Kelso, 2000; Newell, 1996; Thelen & Smith, 1994; van Gelder, 1998).

Co-evolution of motion and object, we further propose, suggests the pedagogical merit of indirect instruction in embodied–interaction learning environments. Indirect instruction, we will argue, provides students the time, space, and license to adapt their intrinsic dynamics (Kostrubiec, Zanone, Fuchs, & Kelso, 2012) by discovering and refining new sensory orientations toward the action field. As we will explain, these action-oriented sensory constructions of the environments are called “attentional anchors” (Hutto & Sánchez-García, 2015).

In this paper we will not adjudicate among direct and indirect approaches to the instruction of new movement. In fact, the rationale of this paper is that future empirical comparisons are predicated on prior theoretical analysis, as we attempt to offer here.

### Objective: taxonomy of movement in embodied design for mathematics learning

The objective of this paper is thus to offer a conceptual analysis of what we all might mean when we talk about movement in embodied-interaction mathematics learning environments. Our proposed taxonomy will implicate three types or facets of movement: (1) distal movements we ultimately effect in the world via mediating instruments; (2) proximal physical movements that handle the instruments; and (3) sensorimotor operatory schemes that organize the performance of these tasks. The instruments we wield to extend our proximal movements may themselves constitute vital elements of mathematical learning and reasoning. When these instruments are immaterial ways of seeing the world, we will call them “attentional anchors” (Hutto & Sánchez-García, 2015) and explain their subjective evolution as problem-solving psychological structures serving situated activity and potentially coalescing as mathematical objects.

As a context for this analysis, we will discuss a form of instructional design inspired by embodiment theory that looks to create conditions for students to develop new proto-mathematical sensorimotor schemes in the absence of direct instruction. At its broadest, the paper is motivated by the assumption that evaluating any plausible pedagogical methodology might, at the very least, enrich the field’s knowledge about cognition and instruction (Easterday, Rees Lewis, & Gerber, 2016). However, it may ensue that this instructional methodology bears advantages for practice.

We believe that students can and should learn to move in new ways through active exploration. Solving dynamical interaction problems rather than being taught directly how to move, we maintain, enables individual students to develop sensorimotor schemes appropriate to their idiosyncratic enactive skill (Chow, Davids, Button, & Renshaw, 2016; Chow et al., 2007). Through subsequent activities of our design, students describe these motor-actions and situated perceptions in the form of mathematical entities (Howison, Trninic, Reinholz, & Abrahamson, 2011). That is why we say that students first learn to move in new ways and then learn mathematics by modeling these new ways of moving (Abrahamson & Sánchez-García, 2016).

Herein, we contextualize our theoretical arguments for conceptualizing movement as sensorimotor activity and considering the qualities of minimally guided instruction. Specifically, we offer a thought experiment elucidating our view of movement. We then offer results from an empirical study suggesting the merits of our proposals.

## Learning is moving in new ways: designing for the emergence of proto-mathematical sensorimotor schemes

What is movement in relation to concepts such that we can design for conceptual learning? This theoretical section offers to unpack the idea of movement in ways that may prove useful for educational designers and, more broadly, for educational scholarship. The section below proposes to split movement into proximal and distal components, and we then explain how technology mediates proximal and distal movements as well as how this mediating role can be leveraged in designing for mathematics learning.

### Raising the question of design: proximal versus distal movement

When we say that students participating in designed educational activities learn mathematical content by first learning to move in a new way, we must clarify the mediating role of technology in acculturating this capacity to move in a new way. Very often, our physical actions are in direct contact with the objects we manipulate, such as when we lift a ball and throw it. Some educational designs leverage these naturally available, unmediated manipulation processes as sources of learning. For example, students may learn about physics principles of kinematics by experiencing and reflecting on the greater physical effort demanded in throwing a ball across greater spatial distance. Such design is both governed and limited by universal laws. However, our contact with the objects we manipulate can be moderated by intervening tools, such as when we use physical utensils (e.g., a fork) or electronic appliances (e.g., a remote control) to extend, augment, distribute, scale, and transform our physical actions over and through space, time, media, cultural forms, and fellow participants (Hutchins, 2014; White, 1984). As such, technology creates enactive distance between intention and effect. Learning to control the environment in instrumented situations is the process of removing this enactive distance by assimilating and mastering the instruments (Morgan & Kynigos, 2014; Pratt & Noss, 2010; Vérillon & Rabardel, 1995). For example, the blind and visually impaired learn to navigate space using a cane, where the cane becomes through practice a sensorimotor extension of the body into a thus expanded “enactive landscape” (Kirsh, 2013).

This principle of sensorimotor augmentation is well known among cognitive scientists of neuroplasticity who build sensory substitution technological systems (Bach-y-Rita, Collins, Saunders, White, & Scadden, 1969). Similarly, designers of educational activities capitalize on this neuroplasticity principle (Siu, 2016). Following principles of the embodied design framework, we build tools whose operatory function is engineered specifically so as to demand, and therefore cultivate, the development of particular sensorimotor schemes as a condition for masterful control of the environment in accord with task demands. In so doing, we target specific sensorimotor schemes that, through instructional guidance, will come to ground the mathematical concepts we want these students to learn (Abrahamson, 2014; Abrahamson & Trninic, 2015).

When a tool mediates an individual’s action on the environment, we often witness differences between proximal and distal action, that is, between the hand motions of manipulating the tool and the extended result of this manipulation in the domain of action. For example, compare the bimanual motion of operating a pair of gardening shears and the effect of these mechanically mediated motions on a branch. Notably, competent shearing is oriented on the branch, not on the hands, unless we experience physical or mechanical breakdown (Koschmann, Kuuti, & Hickman, 1998). In analyzing the dynamics of these activities and the locus of a student’s attention, it is therefore helpful to clarify whether we are referring to the proximal or distal movements.

Whereas the proximal/distal distinction is quite straightforward and probably uncontested, we worry that the distinction is sometimes obscured in discussions of technological design for embodied–interaction mathematics learning. In particular, we are concerned that insufficient distinction is being made in the literature between, on the one hand, the primary manipulations that students enact and, on the other, the effects of these technologically extended manipulations within the domains of action, whether these be mechanical or virtual media. This lack of distinction between proximal and distal actions, we submit, may implicitly hamper our community’s conversation about learning processes and design principles. For example, the distinction could help us implicate where students attend as their teacher guides their work (Shvarts & Krichevets, 2016).

In the next sections, we aim further to clarify the proximal/distal distinction by complexifying the relation between a person’s sensorimotor orientation toward a situation and the technologically moderated environmental effects of their actions. We will be looking at cases where actions are mediated by a computational platform, specifically at tablet-based activities designed for students to learn through solving interaction problems. The cases were selected to offer a two-stepped salvo, as follows.

The next section, a thought experiment, will treat a dissociation between how we move our hands on the tablet interface and what trace the software generates on the screen as the mediated result of our manual motions. We then present this dissociation as bearing potential for educational design. In the subsequent section we discuss an eye-tracking study of how students solve challenging interaction problems leading to mathematical notions. Drawing on empirical findings, we will argue for a systemic conceptualization of proximal and distal movement as task-driven, situated, distributed sensorimotor schemes oriented on emergent perceptions of the environment.

### Interpolating technology into the agent–environment relation mediates new sensorimotor schemes supporting learning objectives

Consider the following activity involving two hypothetical task scenarios. In both Condition 1 and Condition 2, (1) you are presented with a tablet interactive application; (2) on the screen you see a black circular line; and (3) you are asked to draw a red line on top of the black line. In Condition 1, a “finger painting” task, you use your index finger to trace directly along the existing circle perimeter. As you do so, virtual red paint oozes from under your fingertip to cover the black line. In Condition 2, you cannot trace directly on the black circle. Instead, you are asked to imagine this circle as plotted in a Cartesian space. Now you must move your left hand index up/down along the *y*-axis to the left of the circle simultaneous with moving your right hand index right/left along an *x*-axis below the circle. The application plots red points at spatial locations corresponding to the ordered-pair [x, y] Cartesian intersection of your fingers’ respective measured distances from the origin point. You are thus asked to graph a circle manually.^2^

Whereas the two conditions share a task objective of generating a red circle on top of the black circle, clearly Condition 2 is more difficult that Condition 1. Condition 2, unlike Condition 1, presents you with a problem, and more so if you have never before worked in the Cartesian field. To solve this problem, you must engage in inquiry. You actively explore the new space to discover its embedded functions and determine their utilities relevant to the task objective. In so doing, you enter a cycle of tight, rapid, recursive sensorimotor feedback loops, where you attempt various manipulations, attend to their coinciding instrumented effect, and constantly tune your movement pattern. Perhaps you infer heuristics for action and even articulate them verbally. Through practice, you progressively accommodate your operatory schemes so as to assimilate the tool’s discovered affordances for action. Condition 2 thus presented you with a new constraint that initially impeded your capacity to fulfill the task. However, through figuring out how to cope with this constraint you developed new subjective affordance for the environment (Forman, 1988; Greeno, 1994; Newell, 1986, 1996). You have learned to move in a new way through a representational system that has become central in mathematics, the Cartesian system of perpendicular *x*- and *y*-axes.

In this case, we demonstrated two different ways of generating the same geometrical figure. In a sense, the red circle of Condition 1 is not the same as the red circle of Condition 2. At least, from your subjective perspective, Condition 2 demands of you to develop a new phenomenological and conceptual construction of what a circle is from a mathematical perspective (Abelson & diSessa, 1986; Papert, 1980; Piaget & Inhelder, 1969; Wilensky & Papert, 2010). More generally, the available means of production we have learned to access and use in fulfilling an activity task are instrumental in forming our conceptions of the objects we produce and perceive (Baird, 2004; Chase & Abrahamson, 2015; Meira, 1998; Rosenbaum & Abrahamson, 2016).

As educational designers, we target particular concepts by creating both the task objectives and the means of accomplishing those objectives. The designer’s goal ultimately is not about having a student produce a circle per se but having a student struggle to produce a circle with the given means. In the case of Condition 2, the technological interaction conditions are designed to foster opportunities for students to develop a particular sensorimotor scheme – a bimanual coordinated motor action oriented on a geometrical figure. Still, a new coordination is not yet new mathematical knowledge. For manual know-how to become conceptual know-that, students need to use disciplinary frames of reference to re-describe their own actions (Bartolini Bussi & Mariotti, 2008). In so doing, students shift into disciplinary ways of seeing and talking (Abrahamson, 2009; Bamberger & diSessa, 2003; Sfard, 2002).

In this subsection, we offered a hypothetical study of technologically mediated action. For the purposes of this essay, our objective was to differentiate conceptually between proximal and distal movement insofar as this differentiation bears on the practice and theory of educational design for mathematics learning. As such, we contrasted two technologically mediated activity conditions where the goal distal movement was identical (drawing a red circle on a screen) but the proximal movements were dramatically different (tracing with a finger vs. simultaneously operating two orthogonal axes in a Cartesian field). Whereas the distal movement constituted a necessary task goal, the activity’s educational potential was largely determined by the proximal movement required to generate the distal movement. Thus, a study of mathematics learning from an embodiment perspective should focus on the development of proximal movement, not just its distal product.

In the next section we “recede” into proximal movement to demonstrate that it, too, can be usefully differentiated. Looking at empirical data, we will show that different sensorimotor schemes can achieve the same proximal movement. The case will demonstrate variability, both intra-personal and inter-personal, in how students orient sensomotorically toward objects on the tablet screen so as to generate a particular pattern of hand movements.

## Empirical illustration: embodied design of proportional learning

We are building the argument that educational researchers, and more broadly cognitive scientists, should adopt a more nuanced discourse about movement. We view descriptions of manual movement that focus only on how the hand is moving, for example, analyses of the shape, pace, and morphology of the hand’s kinematic path through space, as failing to capture the sensorimotor schemes generating this movement. Namely, objective descriptions of hand trajectory ignore how the student is orienting to the task and environment so as to develop and produce this movement (Brooks & Goldin-Meadow, 2015; Gallagher & Lindgren, 2015; Lindgren, Tscholl, Wang, & Johnson, 2016; Nathan & Walkington, 2016; Ping & Goldin-Meadow, 2008). Nevertheless, we propose, our pedagogical theorizing and intervention should be attending precisely to these schemes. From an enactivist perspective, these schemes, the student’s actionable relation to the biological–cultural context, are the stuff of cognition and thus constitute the goal and mark of conceptual change. In this section we offer empirical findings as evidence supporting our argument for rethinking movement in mathematics teaching and learning.

### Design background

In the previous sections, we contrasted two scenarios to clarify the distinction between proximal and distal movement as well as direct versus indirect approaches to embodied design. A direct approach was to draw a red circle on top of a black one. An indirect one was to move two hands along an *x*- and *y*-axis in such a way that the same thing happens. We aimed to convince the reader that, while the former scenario would engage students in embodied activity, the latter is more likely to involve them in embodied mathematical activity and thus help them develop sensorimotor schemes that are mathematically relevant to the concept of circle.

In this section we continue by thinking through and illustrating what embodied design for learning about proportion could mean. A direct scenario could be to ask students to point at halfway up a bar, or make a bar twice as long. Again, we maintain such embodied tasks would have little potential for learning about mathematics. In our collaborative work, initiated by the Embodied Design Research Laboratory at Berkeley, we have explored many different designs. An early mechanical design involved a student holding two pulleys that were moved up and down in a particular ratio. From an observer’s perspective, the student was moving her hands in a mathematical proportion. But was she learning? Very little. There was no need for reflection (Trninic, Reinholz, Howison, & Abrahamson, 2010).

**Fig. 1:**
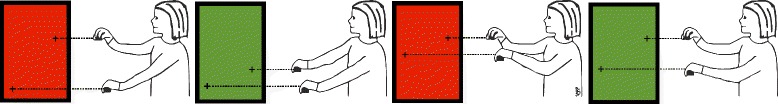
The Mathematical Imagery Trainer for Proportion: schematic activity sequence. The system is here set at a 1:2 ratio, so that the favorable sensory feedback (a *green* background) is activated only when the right hand is twice as high along the monitor as the left hand. This figure sketches out our Grade 4–6 study participants’ paradigmatic interaction sequence toward discovering an effective operatory scheme: (**a**) while exploring, the student first positions the hands incorrectly (red feedback); (**b**) stumbles upon a correct position (*green*); (**c**) raises hands maintaining a fixed interval between them (*red*); and (**d**) corrects position (*green*). Compare b and d, the two green configurations, to note the different intervals between the cursors

The next design iteration was centered on using Wii devices to remote-control two cursors on a screen (Fig. 1). The task was to move the cursors up and down in parallel with the objective of keeping the screen green; this was the case if the cursors’ heights above the bottom of the screen instantiated a particular ratio, regardless of whether the students initially knew or understood this. We call this pedagogical activity architecture a Mathematical Imagery Trainer (Howison et al., 2011).

The Mathematical Imagery Trainer is an interactive technological system designed to foster opportunities for students first to develop targeted sensorimotor schemes and then model these schemes in particular forms that lead to understanding targeted concepts. Now in its tenth year of research, the system has been implemented in a variety of media, including a mechanical apparatus, Wii, Kinect, and iOS touchscreens; it has been evaluated in a variety of settings, including laboratory clinical interviews with individual or paired students and school sites with groups and whole classrooms; and it has served as a context for evaluating an artificially intelligent pedagogical agent. Here, we focus on findings relevant to this theoretical essay. In particular, we will discuss data gathered at Utrecht University suggesting variability in students’ sensorimotor schemes for enacting proximal movement solutions.^3^

### Attentional anchors as mediating proximal and distal movement

The activity task presents students with the motor-action problem of moving their hands in a motion pattern where both hands rise in parallel but at different speeds, for example, the right hand must rise twice as fast as the left hand. (For the sake of readability we will only focus on the 1:2 ratio in the remainder of this paper). We have studied the process by which students learn to move in this new way. Our analyses have drawn on data that include audio–video recordings of students’ actions and multimodal explanations to the researcher, logs of interface actions, and eye-tracking of foveal vision (Abrahamson, Shayan, Bakker, & Van der Schaaf, 2016). These analyses indicated that students were using attentional anchors, as we now explain.

**Fig. 2:**
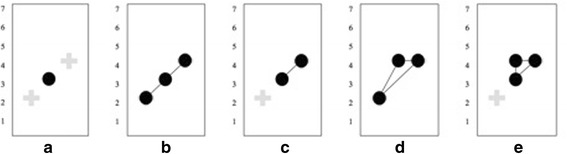
Schematic overview of the variety of emergent dynamical gaze patterns in solving the Parallel Pluses motor-control task. These patterns, which we call attentional anchors, make evident that each student attended to some location between the pluses or at least used that location as a focal gaze point. There is no object to manipulate at that point, in fact, there is no perceptual stimulus there at all. The student constructs and uses the attentional anchor to manage the joint manipulation of both cursors. Patterns **a** through **e** show both intra- and inter-student variability

**Fig. 3:**
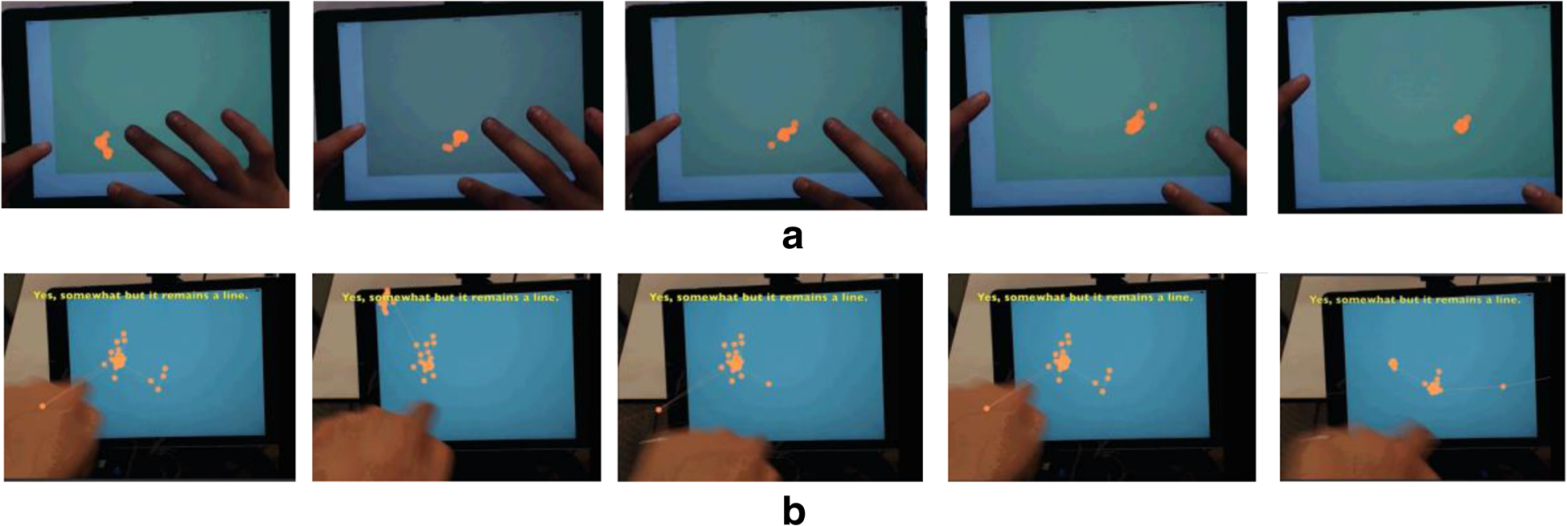
Eye-tracking and clinical data reveal a student’s emergent attentional anchor as their solution to a problem of coordinating bimanual orthogonal movements. In this activity variant, the left hand moves up/down the *y-*axis while the right hand moves right/left along the *x*-axis. The screen will be green only when the left and right hands’ respective distances from the origin (bottom-left corner) relate according to the target ratio (here, 1:2). **a** A student uses an emergent attentional anchor to guide proportional bimanual coordination: they are focusing on an imaginary diagonal line between the tips of their left-hand and right-hand index fingers, keeping this line at a constant angle to the *x*-axis while moving the line to the right. **b** The same student from **a** is explaining their strategy to the experimenter. They gesture an imaginary diagonal line running down from a point on the *y*-axis to a point on the *x*-axis

As students manipulated the two cursors, they attended to particular loci on the screen. These loci included not only the two cursors but also “non-stimuli”, that is, particular locations on the screen where there were no discernable contours (Fig. 2). For example, while moving the two cursors the students would stare at a point on the screen background between the two cursors. To an objective viewer, there was nothing there, and yet the students focused on those points. It appeared that the students were somehow using these loci to facilitate their competent simultaneous manipulation of the two cursors. When students explained their solution strategy in words and gestures, they referred to objects they perceived on the screen, for example, they explained that they were looking at the spatial interval between the two cursors. They would say that this interval should increase as the hands rise and decrease as the hands descend (Fig. 2) or that this interval should move to the right in the Orthogonal Pluses task variant (Fig. 3).

We realized that these imaginary objects emerged into students’ interaction dynamics through the process of solving the control problem of keeping the screen green – the imaginary objects were goal-oriented sensorimotor objectifications of the interaction space. The objects emerged from empty space to help the students perform a challenging motor-control coordination. That is, instead of attending to two hands separately, students could attend to a single object and control it. We further realized that attending to these objects helped students learn the new mathematical ideas, because students referred to these objects in modeling and describing their sensorimotor schemes, first qualitatively and then, once we introduced mathematical tools onto the screen, also quantitatively. Borrowing from Hutto and Sánchez-García (2015), we called these imaginary objects attentional anchors (Abrahamson & Sánchez-García, 2016; Abrahamson et al., 2016).

The empirical context of these studies enables us to track the emergence of a sensorimotor scheme as the integration of two components – a new gestalt in the environment (the “sensori-” component, e.g., a new attentional anchor) and a way of moving relative to this perceptual invariant (the “motor” component, e.g., a new bimanual motion coordination centered on the attentional anchor). In particular, attentional anchors, such as a linear interval between two points, are what we have come to call goal-oriented sensorimotor objectifications. Students construct attentional anchors as their spontaneous solution to motor-control interaction problems. These attentional anchors constitute new proto-mathematical objects amenable to reflecting, modeling, articulating, and expressing in formal symbolic notation. The studies also demonstrate that, whereas light-handed guidance is sufficient for the emergence of attentional anchors, mathematical re-description of these interaction solutions requires more heavy-handed intervention.

In summary, proximal movements may result from a variety of sensorimotor orientations toward the task space – one cannot judge a sensorimotor scheme by its proximal movement cover. In order to understand how students learn mathematics through participating in interaction tasks, it is not enough to describe their distal and proximal movements. We need to dig deeper to find out how they are orienting toward the situation. In particular, more nuanced observation and measurement is necessary for looking under the distal and proximal covers so as to implicate underlying sensorimotor schemes. Educational investigations of embodied learning, we propose, must be geared to theorize, measure, and analyze sensorimotor orientations, as these may constitute the psychological source of proto-mathematical objects. Understanding students’ sensorimotor orientations with the help of attentional anchors should help us better theorize mathematical learning and, in turn, better design mathematics learning systems. For this to occur, educational researchers need to adopt a taxonomy of movement and develop research methods for investigating mathematical moving.

## Conclusion

Whereas researchers informed by embodiment theory generally agree that physical movement plays formative roles in fostering conceptual learning, they have yet to agree over methodology for engaging students in performing these movements (Abrahamson, 2015; Glenberg, 2006; Lindgren & Johnson-Glenberg, 2013; Pouw, van Gog, & Paas, 2014). While some researchers believe we should train students directly to perform the movements, others believe we should let students discover these movements for themselves through working on goal-oriented tasks in appropriately constrained environments. We have proposed that this practical question begs the theoretical question of what we actually mean when we talk about physical movement. Only once we have answered this theoretical question can we, as a community, address problems of practice.

Drawing on both enactivist philosophy and cognitive development psychology, we have argued that externally manifest movement is only the tip of the iceberg, the cusp of deep sensorimotor activity that includes attentional anchors. We demonstrated the utility of using the phrase “proximal movement” to describe physical actions proper and “distal movement” to describe what the proximal movements ultimately enact in the world via instrumental mediation. We further demonstrated that particular proximal movements are themselves consequences of different motor coordination schemes oriented on attentional anchors. It is these sensorimotor schemes, not only the distal or proximal movements, that educational designers are seeking to foster, and we therefore hope our proposed distinctions will prove useful for educational designers. Our insights also suggest a need for more process-oriented studies to understand embodied learning, complementary to experiments in which condition outcomes are compared but in which we know little about the process.

In particular, by rendering sensorimotor schemes conceptually transparent for researchers and practitioners, and positioning these schemes as the cognitive vehicle of mathematical reasoning, we hope to have contributed to a more productive discourse around the potential role of the embodiment approach in educational endeavors. Too often in our anecdotal encounters with colleagues and teachers have we heard what we perceive as under-informed diminutive characterizations of embodied interaction. These views focus on distal movements or perhaps on proximal movement yet with little, if any, concern for the sensorimotor schemes these movements elicit and present with the help of attentional anchors. At the same time, we recognize that much work lies ahead in helping teachers see how students are thinking. Some technological solutions have been put forth as means of making key aspects of sensorimotor schemes manifest as external activity that teachers can access, scrutinize, evaluate, and respond to. Non-invasive co-attention eye-tracking techniques may be one approach (Sharma, Caballero, Verma, Jermann, & Dillenbourg, 2015; Shayan, Abrahamson, Bakker, Duijzer, & Van der Schaaf, 2017; Shvarts & Krichevets, 2016).

Our studies also suggest that, left to their own devices, students each figure out how to coordinate their movements so as to satisfy the interaction task specifications. We recognize the perceived pedagogical tradeoff of students each thinking about a problem in a different way. After all, teachers are mandated to channel students toward normative understanding of curricular concepts. However, it could be that students nevertheless construct situations differently, and that our study only exposed this general phenomenon (Allen & Bickhard, 2013; Kostrubiec et al., 2012). Further, we maintain that both intra-student and inter-student variability in solutions bears developmental benefit for individuals learning to participate in the personal and social enactment of mathematical practices (Abrahamson, Lee, Negrete, & Gutiérrez, 2014). In that sense, our studies demonstrate the cognitive diversity of a collective of students and, in so doing, marks potential for research and practice that leverages this diversity as means of enriching collective argumentation (Abrahamson, Berland, Shapiro, Unterman, & Wilensky, 2006; Asterhan & Schwarz, 2009; Cifarelli & Cai, 2005).

Any discussion of student behaviors and outcomes would be incomplete without attending to the instructors’ actions. We wish to underscore the light-handed approach we have been practicing in our tutorial interviews. This approach to the design and implementation of pedagogical interventions focuses on fostering new ways of moving by managing constraints on action. The approach is coherent with the methodology of teaching experiments (Steffe & Thompson, 2000) and with claims from radical constructivism (von Glasersfeld, 1983, 1992) as well as analogous research on athletic performance (Chow et al., 2016; Chow et al., 2007). As such, empirical research on implementing the constraints-based instructional approach may contribute to current discussions on the process and merit of explorative learning (cf. Kirschner, Sweller, & Clark, 2006). At the least, we have demonstrated the embodied design framework as well as a design architecture, the Mathematical Imagery Trainer, which could issue useful empirical contexts for further investigating individual and interactional mechanisms underlying embodied STEM learning. As interactive technology increasingly enters formal as well as informal STEM education, it should be important for the community of researchers to inform the design and evaluation of consumer products that promise to offer quality embodied learning.

## Footnotes

1 Following Sheets-Johnstone (1999) we conceptualize movement as the source and expression not only of cognition, but also of affective relations with the world; however, in this paper, we focus on cognitive activity.

2 Condition 2 is somewhat comparable to working with an Etch-a-Sketch or its mechanical variants (Nemirovsky et al., 2012).

3 Readers are referred to http://edrl.berkeley.edu/content/kinemathics for a listing of relevant publications. These papers (a) draw on the literature of mathematics education research to explain the rationale and build of the technological system and the range of interaction tasks; (b) report in detail on our methods and results in empirical work with the system; (c) explain why and how students redescribed their sensorimotor schemes mathematically when we introduced symbolic artifacts into the interaction space; and (d) propose contributions for theory, practice, and design. To date, empirical data from studies evaluating the system include behaviors of several hundred students aged 9–14 years.
